# Evaluating the Impact of Environmental Temperature on Global Highly Pathogenic Avian Influenza (HPAI) H5N1 Outbreaks in Domestic Poultry

**DOI:** 10.3390/ijerph110606388

**Published:** 2014-06-19

**Authors:** Zhijie Zhang, Dongmei Chen, Yue Chen, Bo Wang, Yi Hu, Jie Gao, Liqian Sun, Rui Li, Chenglong Xiong

**Affiliations:** 1 Department of Epidemiology and Biostatistics, School of Public Health, Fudan University, Shanghai 200032, China; E-Mails: booew@163.com (B.W.); huy@lreis.ac.cn (Y.H.); agao1224@163.com (J.G.); 1039021808@qq.com (L.S.); mmssddss@gmail.com (R.L.); 2 Key Laboratory of Public Health Safety, Ministry of Education, Shanghai 200032, China; 3 Laboratory for Spatial Analysis and Modeling, School of Public Health, Fudan University, Shanghai 200032, China; 4 Laboratory of Geographic Information and Spatial Analysis, Department of Geography, Faculty of Arts and Science, Queen’s University, 99 University Avenue, Kingston, ON K7L 3N6, Canada; E-Mail: chendm@queensu.ca; 5 Department of Epidemiology and Community Medicine, Faculty of Medicine, University of Ottawa, 451 Smyth Road, Ottawa, ON K1H 8M5, Canada; E-Mail: ychen@uottawa.ca; 6 Department of Microbiology and Health, School of Public Health, Fudan University, Shanghai 200032, China; E-Mail: xiongchenglong@126.com

**Keywords:** avian influenza, highly pathogenic avian influenza, H5N1, environmental temperature, spatial analysis, Bayesian statistics

## Abstract

The emergence and spread of highly pathogenic avian influenza (HPAI) A virus subtype H5N1 in Asia, Europe and Africa has had an enormously socioeconomic impact and presents an important threat to human health because of its efficient animal-to-human transmission. Many factors contribute to the occurrence and transmission of HPAI H5N1 virus, but the role of environmental temperature remains poorly understood. Based on an approach of integrating a Bayesian Cox proportional hazards model and a Besag-York-Mollié (BYM) model, we examined the specific impact of environmental temperature on HPAI H5N1 outbreaks in domestic poultry around the globe during the period from 1 December 2003 to 31 December 2009. The results showed that higher environmental temperature was a significant risk factor for earlier occurrence of HPAI H5N1 outbreaks in domestic poultry, especially for a temperature of 25 °C. Its impact varied with epidemic waves (EWs), and the magnitude of the impact tended to increase over EWs.

## 1. Introduction

Highly pathogenic avian influenza (HPAI) virus subtype H5N1 was first reported on a goose farm in Guangdong Province of China in 1996; human cases were then reported in Hong Kong in 1997 [1,2,3], illustrating for the first time that HPAI H5N1 could be transmitted directly from birds to humans. In late 2003, HPAI H5N1 reemerged and spread from East/Southeast Asia to Central Asia, the Middle East, Europe and Africa. Since then, over 60 countries had experienced HPAI H5N1 outbreaks in domestic poultry and wild bird populations [4]. Although efficient human-to-human transmission has not yet been reported [5], the possibility of transmission among humans cannot be ruled out because of continuous viral mutation and gene re-assortment [6,7]. To efficiently prevent an influenza pandemic from HPAI H5N1, it is important to investigate the risk factors of its occurrence and spread. 

Previous studies have identified the contributions of bird migration, transportation of poultry and poultry products, illegal trade, highway networks, vegetation zones and human activities to HPAI H5N1 outbreaks [8,9,10]. However, there is little evidence so far for direct or indirect impact of temperature on HPAI H5N1 outbreaks [11,12], except for a few indoor experimental studies [13,14,15], in which the temperature was manipulated and kept constant. HPAI H5N1 outbreaks are suspected to be related to environmental temperature that interacts with many other important factors such as bird contact structure [16], host-pathogen interactions (e.g., virulence and immunity), and other environmental conditions [17]. The results from indoor experiments cannot be simply extrapolated to the actual field conditions [18]. To fill the knowledge gap, the evidence is needed for the impact of environmental temperature on HPAI H5N1 outbreaks [19,20]. 

This study was designed to explore the relationships between environmental temperature and global HPAI H5N1 outbreaks in domestic poultry by using an approach of integrating a Bayesian Cox proportional hazards model and a Besag-York-Mollié (BYM) model. We attempted to determine the influences of environmental temperature on global H5N1 outbreaks in domestic poultry on its nature, degree and changes over time. 

## 2. Materials and Methods

### 2.1. Global HPAI H5N1 Outbreaks

Global HPAI H5N1outbreak data in domestic poultry from 1 December 2003 to 31 December 2009 were obtained and integrated from two international organizations—the Food and Agricultural Organization (FAO) and the World Organization for Animal Health (OIE), and other sources (see details in [21]). Epidemic waves were classified with the definition that “during an epidemic wave the number of outbreaks increased rapidly to a peak and then fell more gradually until the epidemic was over”. Six epidemic waves (EW1~EW6) were divided and their number of outbreaks are 2,540, 1,927, 2,338, 657, 682, and 283, respectively, which has been detailed in our previous report [22]. Because of a lack of temperature data for 2009, EW6 that includes outbreaks in 2009 were not included in the present analysis.

### 2.2. Environmental Temperature

Monthly mean air temperature with a spatial resolution of 0.5 by 0.5 degree from December 2003 to December 2008 was obtained from the Center for Climatic Research of the Department of Geography, University of Delaware. Ordinary kriging was first applied to interpolate the monthly temperature to generate sixty-one continuous temperature surfaces (1 + 12 months/years × 5 years = 61); then, the monthly mean temperature for the outbreaks in EW1~EW5 were extracted based on their spatial coordinates and outbreak dates to establish its spatial linkage with global HPAI H5N1 outbreaks [12]. These manipulations were processed using ARCGIS V9.3.1 software (Environmental Systems Research Institute, Inc., Redlands, CA, USA).

### 2.3. Global Polygon Maps

A global administrative map (GADM) with 167,176 administrative areas was used to define the spatial neighbor relationships among HPAI H5N1 outbreaks. It was first spatially joined with H5N1 outbreaks based on their locations using ARCGIS V9.3.1 software and then the adjacency matrix among outbreaks were calculated for EW1–EW5 using the extension tool for determining the adjacency for WinBUGS software developed by Dr. Wayne Thogmartin from the U.S. Geological Survey (USGS). 

### 2.4. Statistical Analysis

Firstly, descriptive statistics were calculated on the environmental temperatures for each EW. The data of global HPAI H5N1 outbreaks in domestic poultry have been described elsewhere [21]. Secondly, smoothing maps of the environmental temperature and the time of outbreak occurrence (outbreak dates) were generated using the technique of ordinary kriging for each EW to depict their patterns of spatial distributions and explore potential relationships. Finally, the integrated approach of a Bayesian Cox proportional hazards model based on the counting process [23] and a Besag-York-Mollié (BYM) model [24] was used to analyze the impact of environmental temperature on HPAI H5N1 outbreaks for each EW. The response variable was outbreak date which was the number of days from the earliest outbreak and the explanatory factor was environmental temperature. Say we have *n* HPAI H5N1 outbreaks. The integrated approach is defined as:

*λ_i_*(*t*)*dt* = *d*Ʌ_0_(*t*)*e^βz_i_^*^ + *u_i_* + *v_i_*^*Y_i_*(*t*) (1)

where, λ*_i_*(*t*) is the intensity process of HPAI H5N1 outbreaks, similar as the mean of a Poisson distribution; *dt* is the size of the time interval:

*d*Ʌ_0_(*t*) = Ʌ_0_(*t*)*dt* (2)

represents the increment of the integrated baseline hazard function of H5N1 outbreaks occurring during the time interval [*t*, *t* + *dt*) and Ʌ_0_(*t*) is a process in which the increment *d*Ʌ_0_(*t*) is distributed as a Gamma distribution; *z_i_* and β indicates the environmental temperature and its unknown regression coefficient, respectively; *u_i_* is the latent variable representing the correlated spatial heterogeneity, which records the synthetical effects from the unmeasured and unknown spatially correlated factors (e.g., precipitation and humidity); *v_i_* is another latent variable and represents the uncorrelated spatial heterogeneity to indicate the comprehensive impact from the unmeasured and unknown spatially independent variables (e.g., control strategies); and *Y_i_*(*t*) is a process taking the value 1 or 0, according to whether the outbreak *i* is observed at time *t* or not. 

For the uncorrelated spatial heterogeneity, the normal non-informative prior was used:

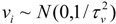
(3)

For the correlated spatial heterogeneity, a spatial correlation structure was used where the risk estimation in any area depended on the neighboring areas. The conditional autoregressive (CAR) model was used here [24,25]:

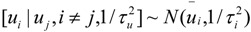
(4)
where: 
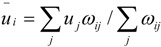
(5)

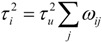
(6) ω*_ij_* = 1 if *i* and *j* outbreaks are adjacent, and 0 if they are not, which are recorded by the adjacency matrix described in Section 2.3.

Bayesian modeling required the specification of prior distributions for unknown parameters, which were all specified by the vague non-informative prior distributions. See the Supplementary Material for a detailed introduction on this integrated approach and specifications of prior distributions for all parameters.

## 3. Results

### 3.1. Environmental Temperature in Different Epidemic Waves

Table 1 shows that HPAI H5N1 outbreaks in EW1 and EW3-EW5 had similar temperature features. Temperature for the outbreaks in EW2 displayed different traits, which had a higher minimum, higher quartiles and a lower maximum temperature, and smaller variations indicated by standard deviation and 95% confidence interval when compared with outbreaks in other EWs. 

**Table 1:** Summaries of environmental temperature for five epidemic waves (EWs).

EW	Sample size	Mean	*std*	*CI*95	Median	Minimum	Maximum	Q1	Q3
1	2,540	20.832	5.615	(8.777, 27.303)	20.140	−11.230	31.525	16.961	26.441
2	1,927	27.178	1.972	(21.177, 29.374)	27.641	6.344	30.679	26.726	28.392
3	2,338	17.578	9.158	(−3.100, 31.180)	17.949	−11.360	34.214	14.538	22.483
4	657	19.742	8.512	(1.563, 30.129)	21.418	−12.990	33.680	14.274	26.504
5	682	18.804	6.767	(1.074, 28.864)	19.279	−7.417	33.632	14.279	24.622

Notes: Q1 and Q3 are the first and third quartile, respectively; *std* means standard deviation; *CI*95 is the 95% confidence interval.

### 3.2. Spatial Distributions of Environmental Temperature and Outbreak Dates

**Figure 1:**
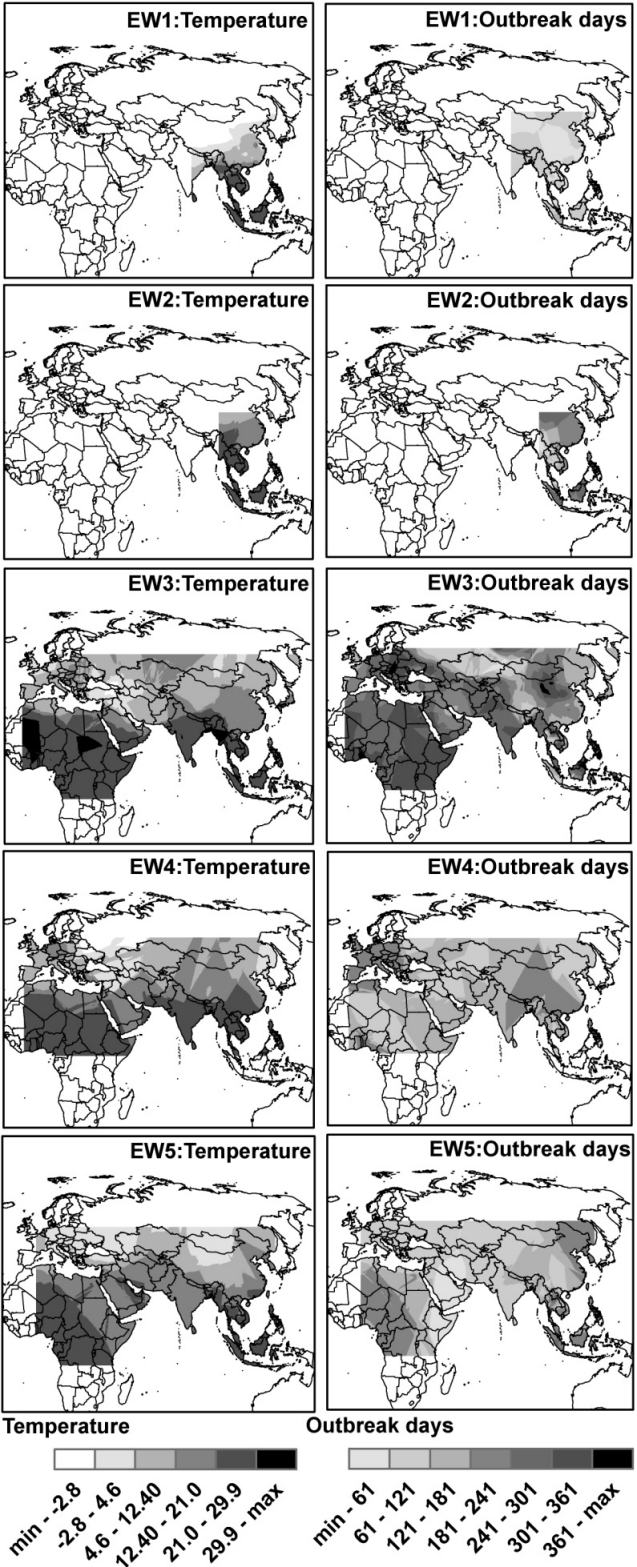
Spatial distributions of environmental temperature and outbreak dates of global HPAI H5N1 outbreaks in domestic poultry across five epidemic waves. The darker the color, the higher the temperature and the later the outbreak dates.

Figure 1 shows that the average environmental temperature for HPAI H5N1 outbreak dates in different EWs was consistently higher in the southern part than in the northern part, which is consistent with the general temperature pattern. However, the pattern of outbreak dates varied substantially across EWs. In EW1 and EW2, outbreaks seemed to spread from the middle part to both sides of south and north; in EW3, outbreaks seemed to disperse westward; while in EW4 and EW5, outbreaks seemed to first appear in the middle part and then spread both east and west (Figure 1).

### 3.3. Impact of Environmental Temperature on HPAI H5N1 Outbreaks

Table 2 summarizes the results from the integrated approach.

There was a significant effect of environmental temperature on HPAI H5N1 outbreak dates in domestic poultry for all five EWs. We observed that the regression coefficients for environmental temperature were all located outside the 95% Bayesian credible interval (BCI). Temperature was negatively related with outbreak dates for EW1 and EW3- EW5, and its (absolute) magnitude showed a tendency of increasing over EWs. However, the temperature of outbreaks in EW2 showed a positive impact on outbreak dates. 

Considering the two components of correlated and uncorrelated spatial heterogeneities after removing the effects of environmental temperature on HPAI H5N1 outbreaks, the impact of the uncorrelated heterogeneity was consistently stronger than that of the correlated heterogeneity. Their squared ratios (variance ratio) were 1.55 in EW1, increased to 3.59 in EW2, peaked in EW3 (73.96) and decreased in EW4 (18.02) and EW5 (19.27).

**Table 2:** Posterior summaries for the analysis results of the integrated approach.

EW	Node	Term	Mean	*std*	MC Error	2.50%	Median	97.50%
1	beta	temperature	−0.023	0.006	0.000	−0.033	−0.023	−0.011
	sigma.u	*std* of u[]	0.069	0.017	0.001	0.043	0.067	0.110
	sigma.v	*std* of v[]	0.086	0.021	0.002	0.051	0.083	0.134
2	beta	temperature	0.232	0.019	0.002	0.198	0.230	0.275
	sigma.u	*std* of u[]	0.258	0.056	0.004	0.166	0.252	0.375
	sigma.v	*std* of v[]	0.489	0.047	0.002	0.398	0.488	0.585
3	beta	temperature	−0.088	0.008	0.001	−0.104	−0.088	−0.072
	sigma.u	*std* of u[]	0.190	0.095	0.007	0.063	0.172	0.421
	sigma.v	*std* of v[]	1.634	0.085	0.004	1.472	1.633	1.806
4	beta	temperature	−0.185	0.015	0.001	−0.217	−0.184	−0.156
	sigma.u	*std* of u[]	0.359	0.271	0.010	0.077	0.279	1.070
	sigma.v	*std* of v[]	1.524	0.179	0.009	1.208	1.511	1.907
5	beta	temperature	−0.134	0.012	0.001	−0.157	−0.134	−0.112
	sigma.u	*std* of u[]	0.177	0.085	0.004	0.066	0.161	0.383
	sigma.v	*std* of v[]	0.777	0.090	0.004	0.614	0.773	0.967

Note: *std* means standard deviation.

## 4. Discussion and Conclusions

Gilbert *et al.* [26] and Curseu *et al.* [11] discussed the possible impact of climate warming on HPAI H5N1 virus and outbreaks in broad and general ways. Liu *et al.* showed that environmental temperature dropped shortly before HPAI H5N1 outbreaks in (live) wild birds and domestic poultry of Eurasia between 2005 and 2006 [20], but only descriptive analysis was applied. Reperant *et al.* found that (daily) maximum surface air temperatures was the most significant risk factor for HPAI H5N1 outbreaks in wild birds in Europe during the winter of 2005–2006, but confounding factors were not adjusted [27]. Si *et al.* concluded that minimum air temperature in January was negatively related with HPAI H5N1 outbreaks in wild birds of Europe from 2005 to 2008 with some anthropogenic and physical environmental factors controlled [19], but the air temperature in their study were from the database before 2000, resulting in biased results because of wrong matching between temperature dates and outbreak dates. So far, however, there has been no reports evaluating the impact of environmental temperature on global HPAI H5N1 outbreaks in domestic poultry. There are some methodological challenges. 

It is important to control potential confounders in order to accurately explore the specific impact of environmental temperature on HPAI H5N1 outbreaks. Traditional methods allow us to adjust for known and measured covariates, which is often infeasible either because of data unavailability or limited knowledge on disease transmission mechanisms. In addition, spatial autocorrelation always exist according to Tobler’s “First Law of Geography”: “Everything is related to everything else, but near things are more related than distant things” [28]. It is crucial to account for the phenomenon of autocorrelation during the analysis. To deal with these issues, we adopted the idea of the Besag-York-Mollié (BYM) model and used two latent variables (components) to represent the correlated and uncorrelated heterogeneities from all the unmeasured and unknown factors that are related to study outcomes, respectively [24]. The former component captures the integrated effects of spatially clustered factors and also can take into account the spatial autocorrelation; while the latter component combines the influences of spatially independent variables. In the current study, because we used the outbreak date (time of outbreak occurrence) as the response variable, we applied the Bayesian Cox proportional hazards model based on the counting process. The approach of integrating the BYM model and Bayesian Cox proportional hazards model allowed us to adjust for the potential impact of unavailable or unmeasured factors through introducing two extra latent variables when we studied the specific impact of environmental temperature on HPAI H5N1 outbreaks in domestic poultry. 

By using the integrated approach of BYM model and Bayesian Cox proportional hazards model, we found that environmental temperature was significantly associated with the dates of HPAI H5N1 outbreaks, either negatively or positively. For all the EWs except for EW2, we found that the higher the environmental temperature, the earlier the dates of HPAI H5N1 outbreaks, suggesting that high environmental temperature increased the risk of earlier occurrence of HPAI H5N1 outbreaks in domestic poultry. This may prompt that climate warming may have an effect of resulting in the earlier arrival of global HPAI H5N1 outbreaks. There were a trend of stronger linkage between environmental temperature and the date of HPAI H5N1 outbreaks over the EWs. We speculate that implemented control measures on HPAI H5N1 outbreaks might reduce or eliminate the effects of controllable risk factors and the impact of environmental temperature became increasingly marked. There was an exception, however. For EW2, the data showed that the higher the environmental temperature, the later the date of HPAI H5N1 outbreaks. We noticed that the features of environmental temperature in EW2 were different from the other EWs (*F* = 590.75, *p* < 0.05). The average environmental temperature in EW2 (27 °C) was much higher compared with other EWs (18 to 21 °C). There is a possibility that environmental temperature may have different impact on HPAI H5N1 outbreaks depending on the level of temperature. We further divided the environmental temperature in EW2 into three levels (≤20, 20–25 and ≥25 °C) and re-analyzed the data using the same approach (Table 3) and found that in the intervals of ≤20 °C and 20–25 °C an increase in environmental temperature was significantly associated with the earlier occurrence of HPAI H5N1 outbreaks, which was different in the interval of ≥25 °C. 

**Table 3:** Posterior summaries on the model parameters of the transformed data in EW2 with environmental temperature divided into three intervals.

Node	Term	Mean	*std*	MC error	2.50%	Median	97.50%
beta[0]	[20,25)	−1.634	1.451	0.122	−3.783	−1.843	1.286
beta[1]	[min,20)	−1.461	0.406	0.007	−2.285	−1.450	−0.689
beta[2]	[25,max]	0.841	0.107	0.004	0.628	0.843	1.047
sigma.u	std of u[]	0.253	0.053	0.003	0.155	0.250	0.361
sigma.v	std of v[]	0.484	0.046	0.002	0.396	0.484	0.577

Note: *std* means standard deviation.

After removing the effects of environmental temperature, the component of uncorrelated heterogeneity showed a consistently stronger impact on outbreak dates compared to the component of clustered heterogeneity. And their variance ratios increased from EW1, peaked in EW3, and then decreased, which seems to be consistent with the overall epidemic situation of global HPAI H5N1 outbreaks. In practice, it is the case that either clustered or uncorrelated heterogeneity dominates the other [24]. If clustered heterogeneity is dominant, then the spatial structure contributed to most of disease risks; otherwise, the disease risks were mainly from the spatial independent factors. For HPAI H5N1 outbreaks, our results may suggest that the spatially uncorrelated risk factors were the main reasons for HPAI H5N1 outbreaks in all EWs, which should be the focus for effective control strategy (e.g., biosecurity measures). 

It is debatable that which temperature index is better for studying the impact of temperature on HPAI H5N1. Previous experimental studies showed that cold temperature is more important for influenza, and therefore monthly minimum temperature may be better than monthly mean temperature [13,14,15,29]. The monthly minimum temperature in outdoor environment however is generally an instantaneous point value which may be not long enough to affect the HPAI H5N1 outbreaks, while monthly mean temperature implies the concept of duration. We were not able to obtain the information on monthly minimum temperature for the study period. Otherwise, a comparison would be useful. In this study, we introduced a novel idea of efficiently analyzing the specific impact of environmental temperature on HPAI H5N1 outbreak dates in domestic poultry. We found that high environmental temperature was a significant risk factor for earlier occurrence of HPAI H5N1 outbreaks in domestic poultry, especially under the temperature of 25 °C. Its impact varied not only with temperature ranges but also with EWs, and the magnitude of the impact showed a tendency of increasing over EWs. In addition, the analysis suggested that spatially independent risk factors (e.g., biosecurity measures) were the main force for HPAI H5N1 outbreaks in domestic poultry. 

## Supplementary Files

Supplementary File 1 Supplementary Information (PDF, 152 KB)
