# Supplementary material for: Evaluating the Impact of Environmental Temperature on Global Highly Pathogenic Avian Influenza (HPAI) H5N1 Outbreaks in Domestic Poultry
Source: Int J Environ Res Public Health. 2014 Jun 19;11(6):6388–99. doi: 10.3390/ijerph110606388 (PMC4078585; doi:10.3390/ijerph110606388)
Supplement: Supplementary File 1 — Supplementary Information (PDF, 152 KB) [file ijerph-11-06388-s001.pdf]

Supplementary Information

Evaluating the Impact of Environmental Temperature on Global Highly Pathogenic Avian Influenza (HPAI) H5N1 Outbreaks in Domestic Poultry

---

The Integrated Approach of Bayesian Cox Proportional Hazards Model and Besag-York-Mollié (BYM) Model

Clayton [1] formulated the Cox proportional hazards model using the counting process introduced by Andersen and Gill [2] and also discussed the approach of Bayesian estimation using Markov Chain Monte Carlo (MCMC) method. Say we have n HPAI H5N1 outbreaks. Let $N_i(t)$ (i = 1,..., n) count the number of outbreaks which occurred up to time t. Then $N_i(t)$ can be regarded as a counting process with random intensity process $\lambda_i(t)$ [1,2]:

$$\lambda_i(t) = \lambda_0(t)e^{\beta z_i}Y_i(t) \quad (i = 1,..., n) \tag{1}$$

where, $\lambda_0(t)e^{\beta z_i}$ is the familiar Cox proportional hazards model: $\lambda_0(t)$ represents the underlying baseline hazard function, β is the unknown regression coefficients, and z_i is the observed or measured covariates for the ith outbreak; $Y_i(t)$ is a process taking the value 1 or 0 according to whether or not outbreak i is observed at time t.

For HPAI H5N1 outbreaks, we have data D= $N_i(t)$, $Y_i(t)$, environmental temperature z_i , unknown regression parameter β and:

$$\Lambda_0(t) = \int_0^t \lambda_0(k)\,dk \tag{2}$$

Then the joint posterior distribution of Bayesian estimation for model (1) is:

$$P(\beta, \Lambda_0(t) \mid D) \sim P(D \mid \beta, \Lambda_0(t))P(\beta)P(\Lambda_0(t)) \tag{3}$$

Assuming non-informative censoring, the likelihood of the data ($P(D \mid \beta, \Lambda_0(t))$) is proportional to l, where:

$$l = \prod_{i=1}^{n}\left(\prod_{t\geq 0}\lambda_i(t)^{dN_i(t)}\right)e^{-\lambda_i(t)dt} \tag{4}$$

This is as if the counting process increments $dN_i(t)$ in the time interval [t, t + dt) are independent Poisson random variables with means $\lambda_i(t)dt$:

$$dN_i(t) \sim Poisson(\lambda_i(t)dt) \tag{5}$$

Following formula (1), $\lambda_i(t)dt$ can written as:

$$\lambda_i(t)dt = d\Lambda_0(t)e^{\beta z_i}Y_i(t) \tag{6}$$

where, a vague Normal prior distribution was used for regression coefficient β (~N(0, 0.0001)):

$$d\Lambda_0(t) = \Lambda_0(t)dt \tag{7}$$

is the increment of the integrated baseline hazard function occurring during the time interval [*t*, *t* + *dt*). Since the conjugate prior for the Poisson mean is the Gamma distribution, it would be convenient if $\Lambda_0(t)$ is a process in which the increment $d\Lambda_0(t)$ is distributed as Gamma distribution. So, the increment was assigned the vague Gamma prior distribution as suggested by [3], namely:

$$d\Lambda_0(t) \sim Gamma(cd\Lambda_0^*(t), c) \tag{8}$$

where, $d\Lambda_0^*(t)$ is a prior guess of $d\Lambda_0(t)$; *c* represents the degree of confidence in that guess and small values of *c* correspond to weak prior beliefs. In this study, we set $d\Lambda_0^*(t) = rdt$ (9) (*r* is a guess at the failure rate per unit time; *dt* is the size of the time interval). The vague Gamma prior distribution (G (0.01, 0.01)) was used for both *c* and *r*.

In our study, there are many factors that can affect HPAI H5N1 outbreaks, but only environmental temperature z_i was obtained. To efficiently explore the impact of environmental temperature z_i on HPAI H5N1 outbreaks, the effects from the other unobserved and unknown variables that are related with HPAI H5N1 outbreaks should be controlled. However, taking into account all the risk factors in a practical analysis is always impossible either because of the difficulties of obtaining data or our limited knowledge on disease occurrence and transmission. Hence, we adopted the idea of the Besag-York-Mollié (BYM) model to represent all the unmeasured and unknown risk factors using two latent variables.

The BYM model was first introduced by Clayton and Kaldor [4] and further developed by Besag *et al.* [5] It decomposed the position-specific random effects of all the unmeasured and unknown variables into two components: one component is the clustering or correlated heterogeneity, which represents the variables that, if observed, would display substantial spatial structure in that the values for a pair of contiguous zones would be generally much more alike than for two separate zones (e.g., precipitation and elevation) and another component is the uncorrelated heterogeneity that models those intra-area effects that vary in an unstructured way between areas (e.g., gender, age, control strategy). That is, the total effects can be divided as:

$$X_i \sim \alpha + x_i + u_i + v_i \tag{10}$$

where, X_i represents the total effects; α is the impact from the baseline level; x_i is the measured covariates that are known or suspected to be related with the disease (e.g., environmental temperature in this study); u_i and v_i are the surrogates (latent variables) for the clustered and uncorrelated heterogeneities from all the unknown and unobserved covariates, respectively.

For the uncorrelated heterogeneity, the independent normal prior distribution was used:

$$v_i \sim N(0, 1/\tau_v^2) \tag{11}.$$

For the correlated component u_i, a spatial correlation structure should be used, where the risk estimation in any area depended on the neighboring areas. The conditional autoregressive (CAR) model proposed by Besag *et al.* [5] was adopted Lawson *et al.* [6]:

$$[u_i \mid u_j, i \neq j, 1/\tau_u^2] \sim N(\bar{u}_i, 1/\tau_i^2) \tag{12}$$

where:

$$\bar{u}_i = \sum_j u_j \omega_{ij} / \sum_j \omega_{ij} \tag{13}$$

$$\tau_i^2 = \tau_u^2 \sum_j \omega_{ij} \tag{14}$$

$\omega_{ij} = 1$ if *i* and *j* outbreaks are adjacent, and 0 if they are not.

The vague Gamma prior distribution (G (0.01, 0.01)) was used for both $1/\tau_i^2$ and $1/\tau_v^2$, as suggested by Bernardinelli *et al.* [7].

By combining the above two ideas of Bayesian Cox proportional hazards model and BYM model, the integrated approach to analyze the impacts of environmental temperature on HPAI H5N1 outbreaks was defined as:

$$\lambda_i(t)dt = d\Lambda_0(t)e^{\beta z_i + u_i + v_i}Y_i(t) \tag{15}$$

The meanings of the parameters ($\lambda_i(t)$, dt, $d\Lambda_0(t)$, β, z_i, u_i, v_i, $Y_i(t)$) and corresponding prior specifications (β, c, r, τ_i, τ_v) are the same as the above mentioned notations.

WinBUGS version 1.4 (Imperial College and MRC, London, UK) was used to fit the integrated model. One chain Markov Chain Monte Carlo simulation was used for parameter estimation. Model convergence was assessed by visual examination of time series plots of samples, and the inference of the parameters was based on a burn-in of 10,000 iterations after 30,000 iterations.

References

1. Clayton, D. Some approaches to the analysis of recurrent event data. *Stat. Methods Med. Res.* **1994**, *3*, 244–262.
2. Andersen, P.; Gill, R. Cox's regression model for counting processes: A large sample study. *Ann. Statist.* **1982**, *10*, 1100–1120.
3. Kalbfleisch, J.D. Nonparametric bayesian-analysis of survival time data. *J. R. Stat. Soc. B* **1978**, *40*, 214–221.
4. Clayton, D.; Kaldor, J. Empirical bayes estimates of age-standardized relative risks for use in disease mapping. *Biometrics* **1987**, *43*, 671–681.
5. Besag, J.; York, J.; Mollie, J. Bayesian image restoration, with two applications in spatial statistics. *Ann. Inst. Stat. Math.* **1991**, *43*, 1–20.
6. Lawson, A.B.; Browne, W.J.; Rodeiro, C.L.V. *Disease Mapping with WinBUGS and MLwiN*; John Wiley & Sons Ltd.: Chichester, UK, 2003; pp. 123–128.
7. Bernardinelli, L.; Clayton, D.; Montomoli, C. Bayesian estimates of disease maps—How important are priors. *Stat. Med.* **1995**, *14*, 2411–2431.

© 2014 by the authors; licensee MDPI, Basel, Switzerland. This article is an open access article distributed under the terms and conditions of the Creative Commons Attribution license (http://creativecommons.org/licenses/by/3.0/).
